# Disparities in Excess, All-Cause Mortality among Black, Hispanic, and White Veterans at the U.S. Department of Veterans Affairs during the COVID-19 Pandemic

**DOI:** 10.3390/ijerph19042368

**Published:** 2022-02-18

**Authors:** Lilia R. Lukowsky, Claudia Der-Martirosian, Aram Dobalian

**Affiliations:** 1 Veterans Emergency Management Evaluation Center (VEMEC), US Department of Veterans Affairs, Los Angeles, CA 91343, USA; claudia.der-martirosian@va.gov (C.D.-M.); aram.dobalian@va.gov (A.D.); 2 Division of Health Services Management and Policy in the College of Public Health, The Ohio State University, Columbus, OH 43210, USA

**Keywords:** all-cause mortality, COVID-19, pandemic, veterans, racial disparities

## Abstract

Background: From 2019 to 2020, all-cause mortality in the U.S. increased, with most of the rise attributed to COVID-19. No studies have examined the racial disparities in all-cause mortality among U.S. veterans receiving medical care (VA users) at the U.S. Department of Veterans Affairs (VA) during the pandemic. Methods: In the present paper, we conduct a longitudinal study examining the differences in mortality among White, Black, and Hispanic veterans, aged 45 years and older, during the first, full year of the pandemic (March 2020–February 2021). We calculated the Standardized Mortality Rates (SMRs) per 100,000 VA users for each racial and ethnic group by age and gender. Results: The highest percentage increase between the number of deaths occurred between pre- and post-pandemic years (March 2020–February 2021 vs. March 2019–February 2020). For Hispanics, the all-cause mortality increased by 34%, while for Blacks, it increased by 32%. At the same time, we observed that an 18% increase in all-cause mortality occurred among Whites. Conclusion: Blacks and Hispanics were disproportionately affected by the COVID-19 pandemic, leading both directly and indirectly to higher all-cause mortality among these groups compared to Whites. Disparities in the all-cause mortality rates varied over time and across groups. Additional research is needed to examine which factors may account for the observed changes over time. Understanding those factors will permit the development of strategies to mitigate these disparities.

## 1. Introduction

The COVID-19 pandemic led to an increase in all-cause mortality in both the U.S. [1,2,3] and internationally [4]. From 2019 to 2020, all-cause mortality in the U.S. increased by 17.6%, with most of the increase attributed to COVID-19 [5]. Examining the all-cause mortality allows for the estimation of both the direct and indirect effects of the pandemic. Additionally, this approach is independent of COVID-19 tests and questions about the accurate reporting of causes of death on death certificates [6], both of which were widely reported as major concerns for the undercounting of deaths during the first several months of the pandemic.

U.S. veterans are older, sicker, and have lower incomes compared to the general U.S. population [7,8]. Nonetheless, reported excess deaths among veterans were lower compared to the general population [9]. During the early months of the pandemic, while Black and Hispanic veterans were more likely to test positive for COVID-19 and to be hospitalized with severe COVID-19 symptoms compared to White veterans, 30-day mortality rates for those who tested positive for COVID-19 were lower for these groups than among White veterans [10,11]. In contrast, the increase in COVID-19 and all-cause mortality among Blacks and Hispanics was higher compared to Whites, in the general population during the same period [6,12]. These studies provide an important insight regarding racial/ethnic patterns of mortality among COVID-19 positive patients, and highlight differences between veterans receiving healthcare services (VA users) at the U.S. Department of Veterans Affairs (VA) and the general U.S. adult population.

To our knowledge, however, no study has comprehensively examined racial disparities in excess, all-cause mortality among U.S. veteran VA users during the pandemic. We conducted a longitudinal study examining differences in mortality among White, Black, and Hispanic Veterans, aged 45 years and older, during the first, full year of the pandemic (March 2020–February 2021), thus allowing us to understand how racial disparities may have changed as societal responses to the pandemic evolved over time. We hypothesize that all-cause mortality trends among different racial and ethnic groups of VA users would follow similar patterns as the U.S. general population.

## 2. Materials and Methods

Using VA administrative and clinical data from the Corporate Data Warehouse (CDW), we created a study cohort consisting of 5.1 million White, Black, and Hispanic VA users aged 45 or older, who accessed healthcare services at the VA at least once between March 2018 and February 2021. We calculated the number of deaths for three consecutive periods: March 2018–February 2019 (period 1), March 2019–February 2020 (period 2), and March 2020–February 2021 (period 3). These periods were chosen based on the start of the COVID-19 surge in the U.S. (March 2020), to compare two years prior to the surge with the first year of the pandemic. We examined the total number of deaths and changes in mortality rates across periods 1, 2, and 3 among each group. Additionally, we obtained the monthly death counts for the same three study periods. The death counts were obtained from the CDW database that included deaths from the VA Death Master File, the Social Security Administration, and other government death certificates [9]. We then calculated Standardized Mortality Rates (SMRs) per 100,000 VA users for each racial and ethnic group by age in 10-year increments (45–54, 55–64, 65–74, 75–84, 85+) and gender, using all VA users for the same period as a standard population. Additionally, we calculated the excess deaths for each race and ethnicity by subtracting the expected number of deaths obtained from SMR analysis, from the observed deaths for each month. For each month, we calculated the percent change in the number of deaths during period 3 and the average deaths for periods 1 and 2 of the same calendar month. The SAS Enterprise Guide 7.1 (SAS Institute, Cary, NC, USA) was used to conduct the analysis.

The study was conducted according to the guidelines of the Declaration of Helsinki and approved by the VA Greater Los Angeles Healthcare System Institutional Review Board (Project Number: 1616040. Approval date: 8 March 2020).

## 3. Results

During period 3, 190,551, 30,455, and 9657 deaths from all causes occurred among Whites, Backs, and Hispanics, respectively. During period 2, the number of deaths for each group was 161,566 (Whites), 23,117 (Blacks), and 7182 (Hispanics). During period 1, it was 136,228, 20,436, and 6351, respectively. The largest change in all-cause mortality was observed between periods 2 and 3, resulting in a 34% increase in deaths among Hispanics and a 32% increase among Blacks. Between periods 1 and 2, the change in all-cause mortality was considerably lower for both minority groups (13% Blacks, 13% Hispanics). Among Whites, we found an 18% increase in all-cause mortality between periods 2 and 3, and a 19% increase between periods 1 and 2 (Table 1).

During period 3, SMRs per 100,000 were 300, 345, and 256 among Whites, Blacks, and Hispanics, respectively. During period 2, SMRs are 228, 245, and 179, while, during period 1, the rates are 181, 207, and 151, respectively (Figures S1 and S2). This constituted an increase of 32%, 40%, and 43% in all-cause mortality between periods 3 and 2 among Whites, Blacks, and Hispanics, respectively, compared to 26%, 19%, and 18% between periods 1 and 2, respectively (Table 1).

**Figure 1:**
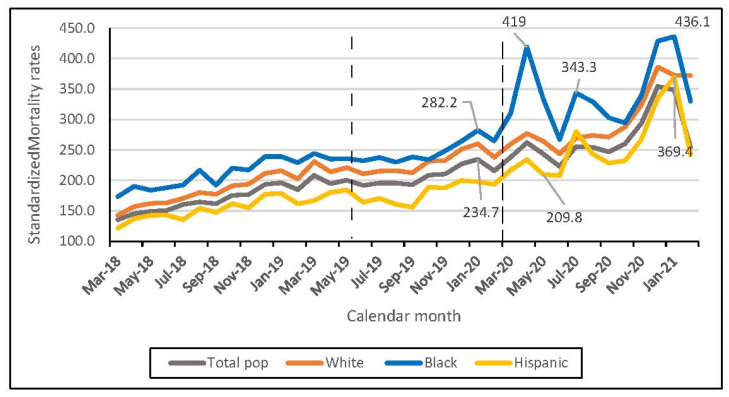
Monthly standardized mortality rates, among White, Black, and Hispanic VA users over 45 years old, between March 2018 and February 2020.

With regard to monthly SMRs, the highest rates are during January 2021 (Whites: 372 per 100,000, 19,690 deaths; Blacks: 436 per 100,000, 3116 deaths; and Hispanics: 369 per 100,000, 1139 deaths), followed by December 2020 (Whites: 386 per 100,000, 20,653 deaths; Blacks: 429 per 100,000, 3063 deaths; Hispanics: 335 per 100,000, 1035 deaths), and April 2020 (Whites: 277 per 100,000, 15,951 deaths; Blacks: 419 per 100,000, 3139 deaths; Hispanics: 234 per 100,000, 763 deaths) (Figure 1 and Figure S3).

**Figure 2:**
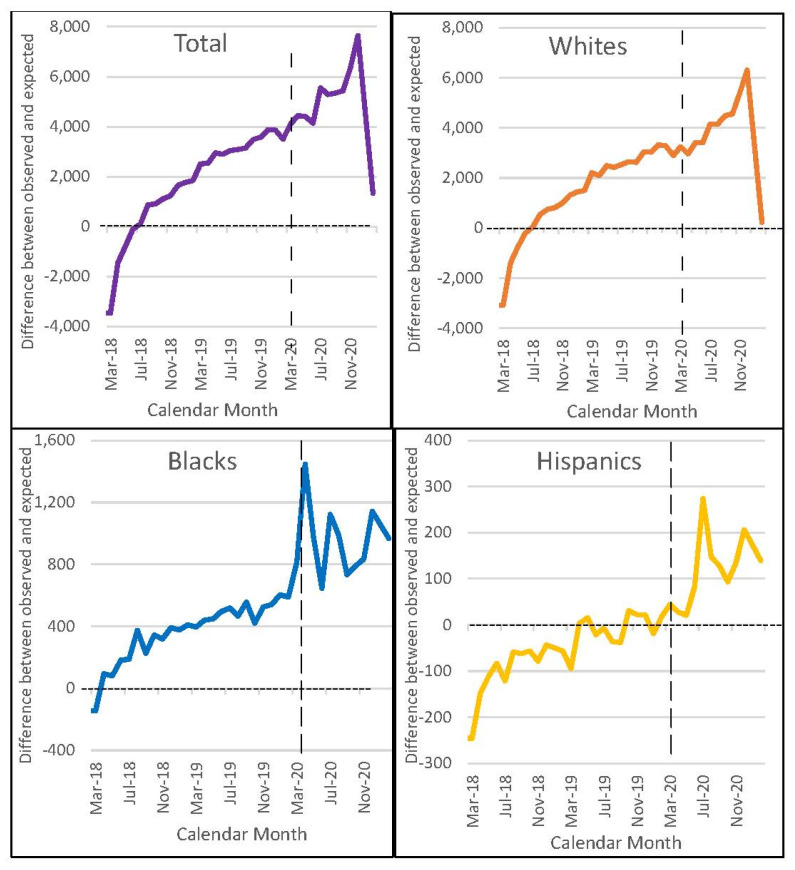
Excess of all-cause monthly deaths as a difference between observed and expected monthly death counts, standardizing by age and gender, among VA users aged 45 years or older by racial/ethnic group from March 2018 to February 2021.

The highest excess all-cause mortality for Whites was observed during December 2020 with 6301 excess deaths. For Blacks, it occurred in April 2020 with 1448 excess deaths, followed by December 2020 with 1143 and July 2020 with 1123 excess deaths. For Hispanics, the highest number of excess deaths was recorded in July 2020 with 274, followed by December 2020 with 207 (Figure 2).

**Figure 3:**
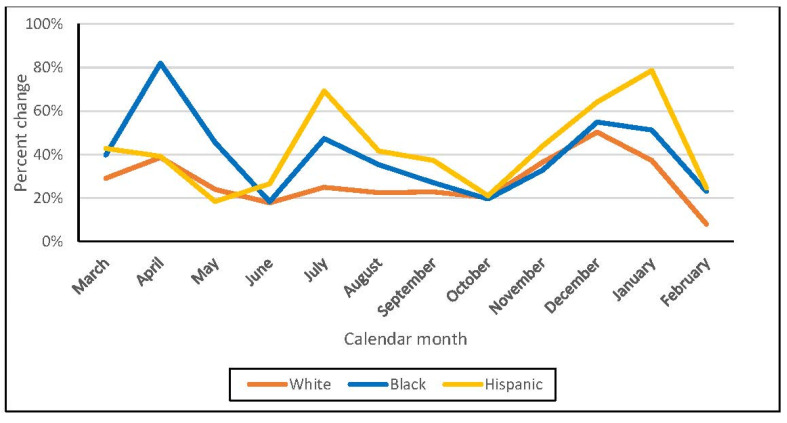
Percent change between each month of period 3 and the average of number of deaths between periods 1 and 2 among VA users aged 45 years or older, between March 2018 and February 2021.

The highest percentage increase between the number of deaths during period 3 and the average deaths for period 1 and 2 for the same month occurred in April 2020 for Blacks (82%). For Hispanics, the highest percent increase in the number of deaths occurs in July 2020 (69%) and December 2020 (79%) (Figure 3).

## 4. Discussion

We observed racial disparities in all-cause mortality among VA users during the pandemic. Our observations were similar to studies examining the U.S. adult population. From period 2 to period 3, we observed a 20% increase in the overall all-cause mortality among VA users aged 45 years and older. We observed a similar 18% increase between periods 1 and 2. Our observations of mortality patterns during periods 1 and 2 followed seasonal variations, with the highest number of deaths occurring between September 2020 and mid-March 2021 (calendar week 35 and week 10, respectively); this is consistent with the trends observed in the general population during the same periods [5].

During period 3, the mortality trends differed from the regular seasonal patterns. Trends during this period occurred in three waves, with the highest number of deaths occurring during December 2020 to January 2021, followed by April 2020, and July 2020. Similar trends were observed in the general U.S. population during calendar year 2020, with the highest number of deaths occurring during the last week of December 2020, followed by week 15 (April 2020) and week 30 (July 2020) [5].

Since most VA users in our study were White, the overall total number of deaths, mortality rates, and changes between periods resemble patterns for White veterans. However, the patterns for Black and Hispanic veterans differ substantially from Whites. The largest increase in all-cause mortality was observed among Black VA users during period 3, which was consistent with observations among Blacks in the general population [6,12]. While Blacks had the highest SMRs during the entire study period, the difference between Whites and Blacks remained relatively consistent during periods 1 and 2. A study conducted before the pandemic found that Black VA users had higher case-specific mortality compared to White VA users [13]. However, during the COVID-19 surge, all-cause mortality among Blacks increased at a higher rate compared to Whites, especially during the three waves in period 3 described above. Higher mortality rates among Black VA users could be due to both direct and indirect factors related to COVID-19. A study conducted by Wong et al. (2021) among a subset of veterans who were tested for COVID-19 between March and November 2020, examined excess mortality at the VA during this period. They found a disparity in spring 2020 among Black VA users, which corroborates the observed increase in all-cause mortality among the same group during April 2020 that we found in this study [14]. Additional factors, such as a heightened risk for obesity, diabetes, cardio-vascular diseases, and high rate of venous thromboembolism (VTE,) among Black Veterans compared to White Veterans [15], combined with decreased hospitalizations at the VA for non-COVID-related causes during the first months of the pandemic [16], likely contributed to the observed increase in all-cause mortality rates among this group, especially during April 2020. Blacks and Hispanics are also more likely to be deemed essential workers and thus were at higher risk of exposure to SARS-CoV-2 compared to Whites. In addition, although the study examined only VA users, it is likely that Blacks and Hispanics, compared with Whites, experience additional barriers that are unrelated to the characteristics of the VA system, but that disproportionately impact their ability to access care.

Other pre-pandemic studies have shown that Hispanics had lower all-cause mortality compared to Whites in the general population [17] and among VA users [13]. We found that the all-cause mortality for Hispanic VA users over 45 years of age was lower compared to Whites during the entire study period. However, the pre-pandemic mortality advantage that existed among Hispanic Veterans compared to White Veterans diminished during COVID-19. This pattern was also evident among the general population, where previous studies have shown that the mortality gap between Hispanics and Whites decreased [12] or was even reversed during the pandemic [17]. The largest increase in all-cause mortality rates among Hispanics occurred during December 2020. Additionally, we observed an increase in all-cause mortality among Hispanic VA users in July 2020. This increase among Hispanics coincided with the increased COVID-19 mortality observed in this group during July 2020 [14].

While all racial and ethnic groups showed an increase in the number of deaths monthly, our results indicate that Hispanics and Blacks had the highest percent changes overall as well as monthly. While Blacks had the largest percent change in all-cause mortality early in the pandemic, Hispanics showed an increase in mortality from June 2020 through January 2021. This finding demonstrates the substantial negative impact of COVID-19 on Hispanics, despite their lower mortality rates compared to Whites. Multiple factors likely contributed to this increase in deaths, including the fact that Hispanics are disproportionately likely to be considered as essential workers for whom adherence to stay-at-home orders and social distancing is more difficult or impossible. Even if the Hispanic Veteran was not employed in such a position, he or she may have other family members who were disproportionately engaged in these types of occupations.

Furthermore, we observed excess mortality during the entire study period for all racial/ethnic groups. However, only Blacks and Hispanics had a rapid increase in excess deaths during period 3, while Whites showed slower and more gradual changes up until November 2020. This suggests that racial and ethnic minority VA users experienced a greater COVID-19 mortality burden compared to White VA users.

One of the reasons for this disparity described in previous studies was the limited access to quality health care among minorities in the general population [18,19]. Since VA is an integrated healthcare system that provides comprehensive healthcare to Veterans, it is somewhat less likely that disparities in access and quality of healthcare played a significant role in the observed mortality disparities between Whites and racial and ethnic minorities. Disparities in access to care tend to be lower among Veterans than in the general U.S. adult population because of factors that include reduced financial barriers [20].

There are several additional reasons that could contribute to the higher mortality burden among Black and Hispanic VA users that are similar to the general population. Blacks and Hispanics, both in the general population as well as at the VA, are more likely to experience a greater burden of comorbidities [18,20,21], which could contribute to higher rate of COVID-19 deaths and complications [22,23,24]. Additionally, and as noted above, household factors, such as a higher percentage of frontline workers and a greater percentage of large, multi-generational households, might also contribute to higher levels of COVID-19 exposure among Hispanics and Blacks [24,25].

Our study has limitations. Compared to the general U.S. population, VA users are older, sicker, have lower incomes, and 90% are male [7,8]. Therefore, our study’s results should not be generalized to the adult U.S. population. However, it should be noted that all-cause mortality trends observed in our study did not differ substantially from the U.S. general population, despite this population being at greater risk for COVID-related mortality because of these sociodemographic factors. Given that minority populations disproportionately reside in urban areas, rurality may have an impact on all-cause mortality trends among the different racial/ethnic groups. However, due to the lack of access to data on patients’ home addresses, we were unable to assess the impact of rurality on all-cause mortality among VA users. Additionally, we did not have information on the causes of death among VA users, as these data are usually released with a lag of 1–2 years. Therefore, we were unable to identify the veterans who died from COVID-19 according to their death certificates, and could not conduct the analysis based on causes of deaths. However, it has been noted that using vital statistics to attribute mortality related to COVID-19 might differ nationwide and may disproportionately affect attribution among racial and ethnic minorities that disproportionately suffer from comorbidities that may incorrectly be identified as the cause of death rather than COVID-19 [26]. There might be a small number of deaths among VA users that were missed if they occurred outside of the VA. However, we used CDW data, a comprehensive database of VA users that captures information in-real time. It contains date of death information obtained from multiple sources, VA Death Master File, Social Security Administration, and other government death certificates, which allowed us to capture the dates of death for this study. Previous studies reported that more than 96% of deaths were recorded in the CDW within 90 days from the day of death [9]. Furthermore, it is possible that declines in non-COVID hospitalizations rates during the first few months of the pandemic might have contributed to excess mortality among VA users. This topic should be explored in future studies to differentiate the direct and indirect impact of the pandemic on mortality.

## 5. Conclusions

Our study contributes to the growing evidence that Blacks and Hispanics were disproportionately affected by the COVID-19 pandemic, leading both directly and indirectly to higher all-cause mortality among these groups compared to Whites. Excess mortality also accounts for the potential disparate effect of delayed care on racial and ethnic minorities who are more likely than Whites to suffer from chronic comorbidities. That delayed care would, in some instances, place them at a heightened risk of death. As our study demonstrates, disparities in mortality rates during the current pandemic varied over time and across groups. Future studies should consider the factors that may account for the observed changes over time. Understanding those factors permits the development of strategies to mitigate these disparities. In particular, studies should examine the variations related to differential state and local government mandates for masks, social distancing, and business closures over time and in relation to racial and ethnic disparities, geographical variations of racial/ethnic composition in urban vs. rural areas, as well as differential access to COVID-19 treatments across racial and ethnics groups and VA facility level adoption rates of telehealth as an alternative to in-person care.

## Figures and Tables

**Table 1:** Number of deaths and percent change in deaths during by period among VA users over 45 years old by racial/ethnic group between March 2018 and February 2021.

	White	Black	Hispanic	Total	White	Black	Hispanic	Total
	Number of deaths				Age, gender Standardized mortality rates per 100,000			
Period 1 *	136,228	20,436	6351	163,015	181	207	151	166
Period 2 *	161,566	23,117	7182	191,865	228	245	179	206
Period 3 *	190,551	30,455	9657	230,663	300	345	256	270
	Percent change				Percent change			
Periods 1, 2	19%	13%	13%	18%	26%	19%	18%	24%
Periods 2, 3	18%	32%	34%	20%	32%	40%	43%	31%

* Period 1: March 2018–February 2019. Period 2: March 2019–February 2020. Period 3: March 2020–February 2021.

## Data Availability

De-identified, aggregated data can be provided upon request.

## Supplementary Materials

The following supporting information can be downloaded at: https://www.mdpi.com/article/10.3390/ijerph19042368/s1.
