# Supplementary material for: Disparities in Excess, All-Cause Mortality among Black, Hispanic, and White Veterans at the U.S. Department of Veterans Affairs during the COVID-19 Pandemic
Source: Int J Environ Res Public Health. 2022 Feb 18;19(4):2368. doi: 10.3390/ijerph19042368 (PMC8874890; doi:10.3390/ijerph19042368)
Supplement: Supplementary file 1 [file ijerph-19-02368-s001.zip › ijerph-1545483-supplementary.pdf]

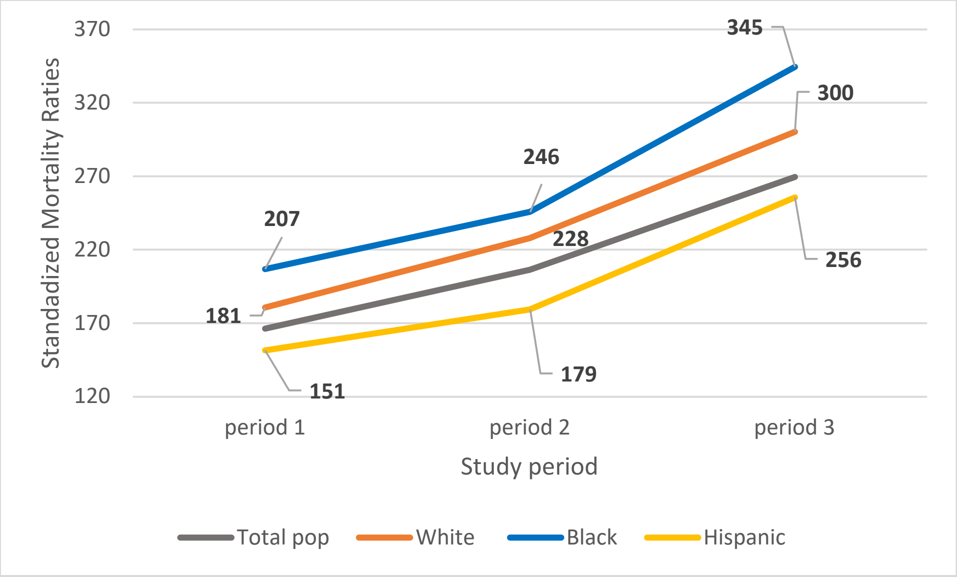

Figure S1. Standardized Mortality Rates for the yearly study periods*, among White, Black, and Hispanic VA users aged 45 years or older, between March 2018 and February 2020

*Period 1 March 2018 – February 2019
Period 2 March 2019 – February 2020
Period 3 March 2020 – February 2021

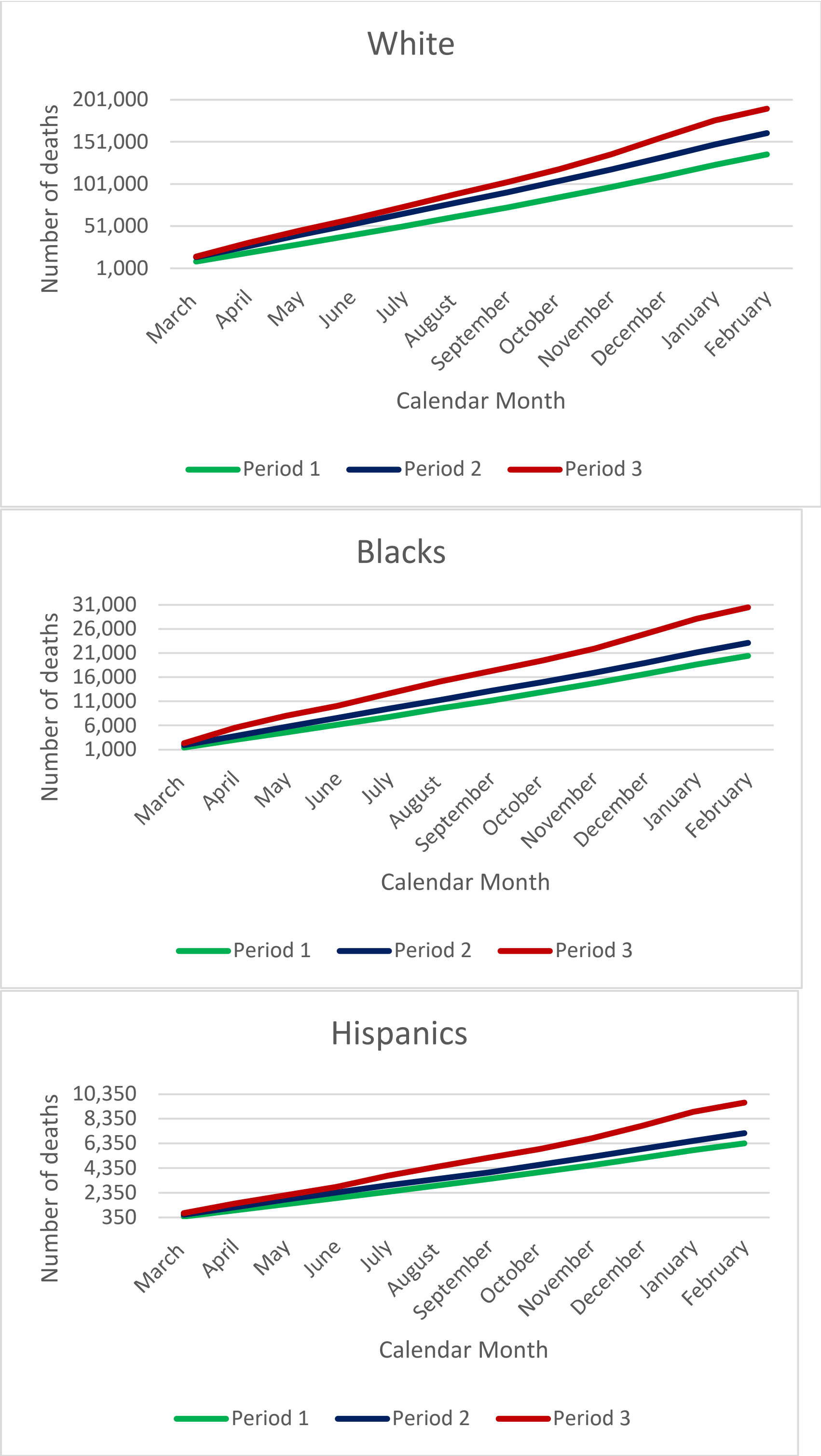

Figure S2. Cumilative death counts per study period* by racial/ethnic group among VA users over 45 years or older, between March 2018 and February 2020.

*Period 1 March 2018 – February 2019; Period 2 March 2019 – February 2020; Period 3 March 2020 – February 2021

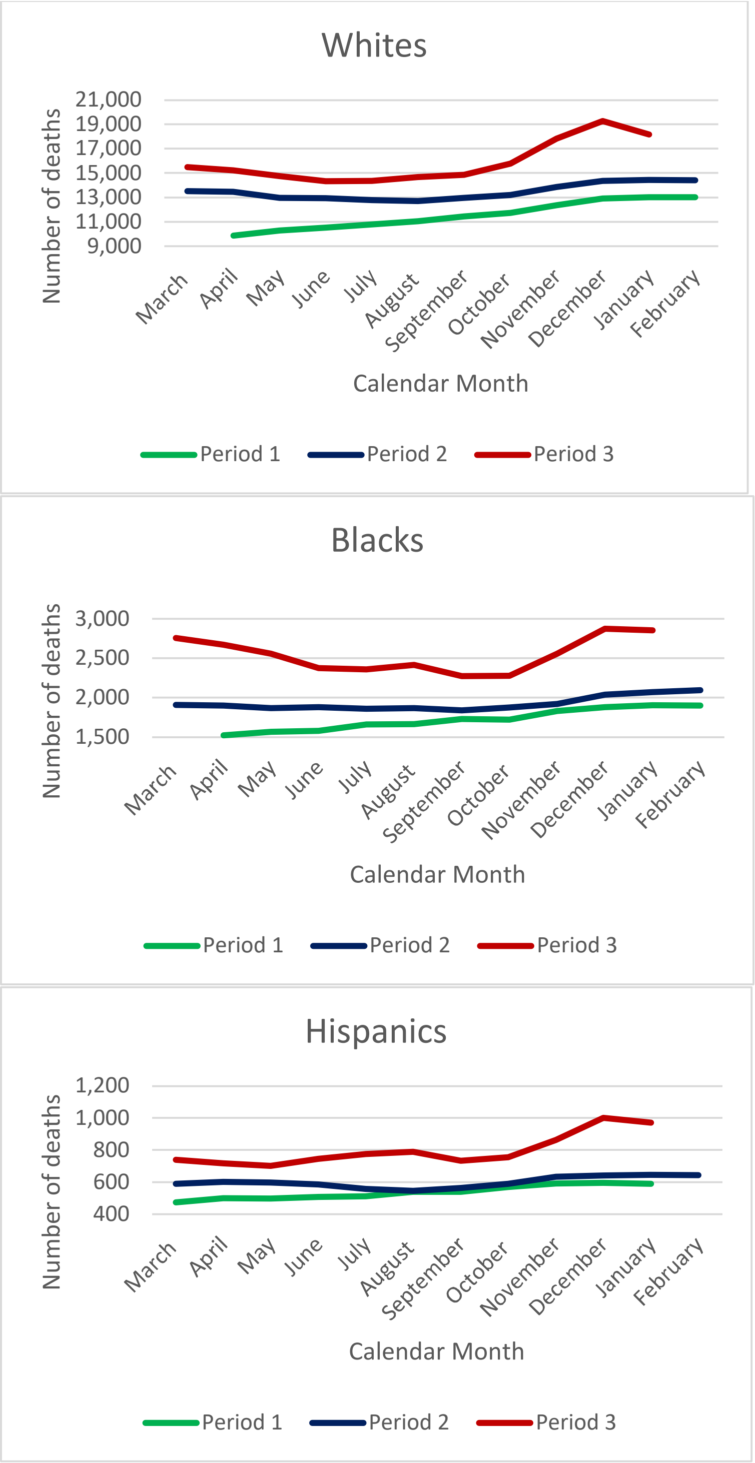

Figure S3. Monthly death counts using 3-momth moving average per study period* by racial/ethnic group among VA users over 45 years or older, between March 2018 and February 2020.

*Period 1 March 2018 – February 2019; Period 2 March 2019 – February 2020; Period 3 March 2020 – February 2021
